# Genetic diversity analysis of Korean peanut germplasm using 48 K SNPs ‘Axiom_*Arachis*’ Array and its application for cultivar differentiation

**DOI:** 10.1038/s41598-021-96074-4

**Published:** 2021-08-17

**Authors:** Rizwana Begum Syed Nabi, Kwang-Soo Cho, Rupesh Tayade, Ki Won Oh, Myoung Hee Lee, Jung In Kim, Sungup Kim, Suk-Bok Pae, Eunyoung Oh

**Affiliations:** 1 Department of Southern Area Crop Science, National Institute of Crop Science, Rural Development Administration, Miryang, 50424 Republic of Korea; 2 Laboratory of Plant Breeding, School of Applied Biosciences, Kyungpook National University, Daegu, 41566 Republic of Korea

**Keywords:** Genetics, Molecular biology, Plant sciences

## Abstract

Cultivated peanut (*Arachis hypogaea*) is one of the important legume oilseed crops. Cultivated peanut has a narrow genetic base. Therefore, it is necessary to widen its genetic base and diversity for additional use. The objective of the present study was to assess the genetic diversity and population structure of 96 peanut genotypes with 9478 high-resolution SNPs identified from a 48 K ‘Axiom_Arachis’ SNP array. Korean set genotypes were also compared with a mini-core of US genotypes. These sets of genotypes were used for genetic diversity analysis. Model-based structure analysis at K = 2 indicated the presence of two subpopulations in both sets of genotypes. Phylogenetic and PCA analysis clustered these genotypes into two major groups. However, clear genotype distribution was not observed for categories of subspecies, botanical variety, or origin. The analysis also revealed that current Korean genetic resources lacked variability compared to US mini-core genotypes. These results suggest that Korean genetic resources need to be expanded by creating new allele combinations and widening the genetic pool to offer new genetic variations for Korean peanut improvement programs. High-quality SNP data generated in this study could be used for identifying varietal contaminant, QTL, and genes associated with desirable traits by performing mapping, genome-wide association studies.

## Introduction

Peanut (*Arachis hypogaea* L.) is one of the important self-pollinated leguminous and oilseed crops cultivated globally. It plays a vital role in human and animal nutrition by serving as a rich source of protein, oil, essential fatty acids, vitamins, and minerals^1^. The current polyploidy peanut cultivar is originated from central South America, natural hybridization between two wild diploids with A and B genome from the polyploidy peanut. Precisely, it is an allotetraploid (2n = 4x = 40, AABB) derived from chromosomal sets belonging to two ancestral species, namely *A. duranesis* and *A. ipaensis* (Genome A, 2n = 20 and Genome B, 2n = 20)^2–5^.

America Archeological studies and evolutionary studies have suggested that peanut has been domesticated roughly 3500 years ago. For centuries it has been used in cultivation and breeding selection^6, 7^. Currently, cultivated peanut has experienced a genetic bottleneck due to the domestication and continuous selection process spanning over centuries. Mainly, reduction in population size could have an impact on genetic variation, secondly relationship between genetic drift and population size also contributed to lower the genetic base in peanut. Therefore, the current cultivated peanut have a very narrow genetic base^8^, although they exhibit good qualitative, quantitative, and agronomic traits^9, 10^. The availability of molecular markers with genetic and physical maps in diploid as well as tetraploid species has facilitated the understanding of the genetic makeup, breeding traits, evolution, and the diversity of genomic resources^11, 12^. Besides, both ancestral species of peanut, namely *A. duranensis* (A subgenome) and *A. ipaensis* (B subgenome), have been sequenced, and the draft genome sequence is publicly available, thus greatly facilitating peanut molecular breeding research^7, 13^.

A precise knowledge of the genetic diversity of cultivated crops is highly crucial for breeding and crop improvement. Thus, additional studies on peanut quality, quantity, and agronomic traits are needed. Several studies on the genetic diversity of cultivated peanut have been performed using different marker systems^14–20^. However, earlier studies have used a relatively limited number of peanut accessions, predominantly SSR markers for evaluations. Therefore, it is necessary to explore the genetic variation and diversity among the cultivated peanut species and the genetic resources available for peanut breeding. Although the SSR markers are co-dominant and used predominantly for detecting genetic diversity and variation among the genotypes or populations, they need allele separation that is a tedious process. Recent advances and the development of next-generation sequencing (NGS) and cost-effective approaches in recent decades have suggested that single-nucleotide polymorphic (SNP) markers are superior to SSR markers and other tools. SNP markers have been widely utilized in diversity analysis, evolutionary studies, association mapping, diagnostics, and fingerprinting, thereby contributing to the success of precise and efficient interpretation of genetic resources and improved breeding. SNPs are by far the most predominant biallelic DNA sequence variations occurring in plant genomes. They can facilitate automation and high throughput assay development^21, 22^. Besides, SNP markers can reduce the error rate in allele calling and enhance the reproducibility of results across laboratories.

Generally, DNA variation in plant genomes is detected using methods such as genotyping-by-sequencing (GBS), whole-genome sequencing (WGS), and high-density fixed arrays^23–25^. These methods have their advantages and shortfalls. For instance, although the GBS method is cost-effective and rapid, it cannot generate allelic data for all SNPs detected between individual genotypes and lines in a subjected population. Besides, the accuracy of this method is affected by abundant missing data. Thus, it requires a well-established setup of bioinformatics pipelines and imputing algorithms, which may not be possible for all laboratories. Conversely, SNP arrays are more expensive than GBS but having high and robust call rates are used to detect SNPs for all the genotypes/lines^26^. This is one of the most important prerequisites for genomic selection. It is the key to consistent genotyping of SNP loci across different individual sets in the population compared to subsequent breeding generations. Therefore, several fixed high-density SNP arrays have been developed for diverse plant species, including peanut (58 K Axiom_*arachis* SNP), rice (Illumina RiceSNP50), maize (MaizeSNP600K Affymetrix Axiom Maize Genotyping Array), soybean (SoySNP50K, 180 K Axiom SoyaSNP), wheat (Axiom Wheat 660 K, 820 K SNP array), chickpea (50 K Axiom CicerSNP Array), cowpea (Illumina 60 K iSelect BeadArray), and pigeonpea (56 K Axiom Cajanus SNP)^13, 27–35^.

In the recent decade, several studies have used different marker platforms including SNP array and GBS to investigate the genetic diversity and population structures of peanuts. For instance, agronomic traits related to domestication have been investigated in 158 Chinese peanut accessions via specific-locus amplified fragment sequencing (SLAF-seq)^36^. Similarly, an ‘Axiom_*Arachis*’ (58 K SNPs) array has been used to assess a reference set of 300 peanut germplasm based on genetic architecture^37^. Recently, one study has explored evolutionary relationships among 320 peanut germplasm using a tunable genotyping-by-sequencing (tGBS) method^19^. Therefore, the relationship between genetic resources can be determined using a high-throughput technology based on abundant SNP variations in the genome. Genetic diversity is critical for crop improvement, germplasm protection, and conservation of diversity in domesticated cultivars. Besides, novel alleles can be detected for future breeding and crop improvement programs. Since the launching of peanut breeding program during the early 80’s in Korea, cultivars have been developed based on a traditional pedigree breeding method by selecting the prominent line merely based on morphological evaluation in the field. Thus, we decided to investigate the genetic backgrounds and determine the genetic pool of peanuts in Korea for the framework of future peanut breeding programs. In addition, we attempted to introduce novel genotypes for enhancing the genetic variations of peanuts.

Thus, the objective of this study was to genotype and interpret the genetic diversities among 96 peanut genotypes, including (varieties and germplasm) widely found in Korea and across the globe using the ‘Axiom*_Arachis*’ 48 K SNPs array. Results reported herein represent the genetic variations among 96 peanut genotypes. Besides, the high-resolution polymorphic SNPs reported in this study can be used to distinguish different peanut cultivars based on Kompetitive Allele-Specific PCR (KASP) markers. These high-resolution polymorphic SNPs can also be used to detect genetic contaminants among the newly released cultivars, create genetic maps, map the desirable traits, and facilitate marker-assisted molecular breeding selection.

## Method and materials

### Plant materials

A total of 96 peanut genotypes were obtained from the National Agrobiodiversity Center, Rural Development Administration (RDA), Republic of Korea (Table S1). These genotypes originated in the following countries: (Argentina-1 (Germplasm), China-5 (Germplasm), India-7 (Variety), South Korea-71 (Germplasm), Taiwan-1 (Germplasm), USA-10 (domestic variety and germplasm), and Zimbabwe-1 (Germplasm). These genotypes were selected based on maturity, yield, other phenotypic characteristics, and the type of botanical variety. These 96 peanut genotypes consisted of 44 var. *hypogae*a, 49 var. *vulgaris*, and 3 var. *fastigiata* (Table S1). The plant meterials used in the present study complies with international, national and/or institutional guidelines.

### Sample collection and DNA isolation

A total 96 genotypes were grown in the National Institute of Crop Science, RDA, R. Korea. Fresh and young leaves collected from 15-day seedlings were almost uniform with the same age for all plants. High-quality genomic DNA extraction was performed using a Quigene DNA kit (Nucleospain, Plant 11-Ref; 740,770.250; Lot No: 1909/001) with minor modifications. DNA quality and quantity were assessed as reported previously^38^.

### Genotyping and SNP allele calling

High-quality DNA of 96 genotypes were used to generate genotyping data with the ‘Axiom_*Arachis*’ 48 K SNP array^37, 39^ containing highly informative genome-wide SNPs (Table S3, S4). The sample SNP genotyping and SNP calling were performed using an Affymetrix GeneTitanplatform as described by Pandey et al.^37^. In brief, target probes were initially prepared for each sample and subjected to amplification, fragmentation, and hybridization on the chip, followed by DNA ligation and signal amplification. Then GeneTitan Multi-Channel Instrument was used for sample staining and scanning. For all 96 peanut samples, allele calling was performed using the software Axiom™ Analysis Suite version 1. To perform the sample quality control (QC) analysis, the ‘*Best Practices*’ workflow was used to select the desired samples that passed the QC test. Further, genotype calls were generated using the ‘*Sample QC*’ workflow, followed by the ‘*Genotyping*’ workflow for genotyping on the imported cell files (http://media.affymetrix.com/support/downloads/manuals/axiom_analysis_suite_user_guide.pdf). Finally, the ‘Summary Only’ workflow was performed to generate a summary and retrieve SNP data. SNPs with low call rates were removed with selection criteria of DishQC (DQC ) > 0.75 and call rate > 90%. Only high-quality SNPs were selected for further analysis.

### Genetic diversity analysis

SNP data were used to elucidate the genetic diversity and the genetic relationship among individual genotypes. In addition, SNP information of public domain US mini-core genotypes was downloaded and merged with the Korean set of genotypes. These sets were then merged and designated as Merge set genotypes to assess the genetic diversity. Genetic diversity parameters such as allele frequency, heterozygosity, and polymorphic information index (PIC) were measured using the PowerMarker version 3.25^40^ and TASSEL version 5.2.39 software with the default setting.

### Population structure

Population structure analysis was conducted using a Bayesian clustering method implemented in STRUCTURE 2.3.4 software^41^. We used K = 1 to 10 to investigate the number of populations (K). Evolution genetic clusters were estimated from the replication runs with the burn-in period up to 500,000 and the Markov chain Monte Carlo (MCMC) to repetitions at each K value. In the STRUCTURE, LnP(D) representing the maximum likelihood can be obtained based on the K value. It is generally considered as an optimal number of a subpopulation^42^.

### Principal component analysis (PCA)

Principal component analysis (PCA) was conducted using TASSEL version 5.2.39 software (https://tassel.bitbucket.io/) using identity-by-state (IBS) matrix data. Pairwise IBS allele sharing was performed using PLINK V1.90 based on multidimensional scaling (MDS) plot^41^.

### Phylogenetic tree analysis

Phylogenetic analysis were performed to obtain insight into the genetic diversity of peanut genotypes. Meanwhile, a phylogenetic tree was constructed using MEGA software (http://www.megasoftware.net/.) with the neighbor-joining method with the following parameters: a test of phylogeny, bootstrap method; no. of bootstrap replications, 1000; model/method, maximum composite likelihood; substitutions to include, d: Transitions + Transversions; and Gaps/missing data treatment, pairwise deletion. A cluster was manually divided into subclades based on the hierarchically clustered genotype profiles.

### Analysis of molecular variance

To examine the molecular variance among and within the populations, an analysis of molecular variance (AMOVA) was performed. Both Korean set and Merge set of peanut genotypes were further analyzed using Arlequin ver. 3.5.2 (http://cmpg.unibe.ch/software/arlequin35) to estimate the pairwise genetic distance (F_ST_) for the subpopulations and to calculate the genetic variation between and within populations.

### Development and validation of KASP markers

KASP markers were developed based on polymorphic analysis of axiom data putative SNPs to distinguish the 17 diverse genotypes currently used in the Korean peanut breeding program and can also be used for varietal identification. Five sets of KASP assay markers were designed by identifying the flanking sequences (50 bp each upstream and downstream) around the SNP position (Table S5). Validation and genotyping were performed for these 17 selected genotypes using the robust KASP assay. The KASP assay amplification and allelic discriminations were performed using a Quant Studio 3Real-Time PCR System (Thermo Fisher Scientific Korea Ltd.). The KASP assays were performed according to the manufacturer’s standard protocol. In brief, 5–10 ng of genomic DNA template was mixed with 5 µL of KASP reaction mixture and 0.14 µL KASP assay. After mixing well, assays were performed with the following thermal cycling conditions: 15 min at 94 °C, a touchdown phase of 10 cycles at 94 °C for 20 s and at 61 °C-55°C (dropping 0.6 °C per cycle) for 60 s, and 26 cycles at 94 °C for 20 s and 55 °C for 60 s (first PCR stage). Recycling was then performed, including three cycles of 94 °C for 20 s and 57 °C for 60 s (second PCR stage). The recycling was performed twice and fluorescence reading was viewed for KASP genotyping after PCR amplification.

## Results

### Selection and genome-wide distribution of selected SNPs

**Figure 1:**
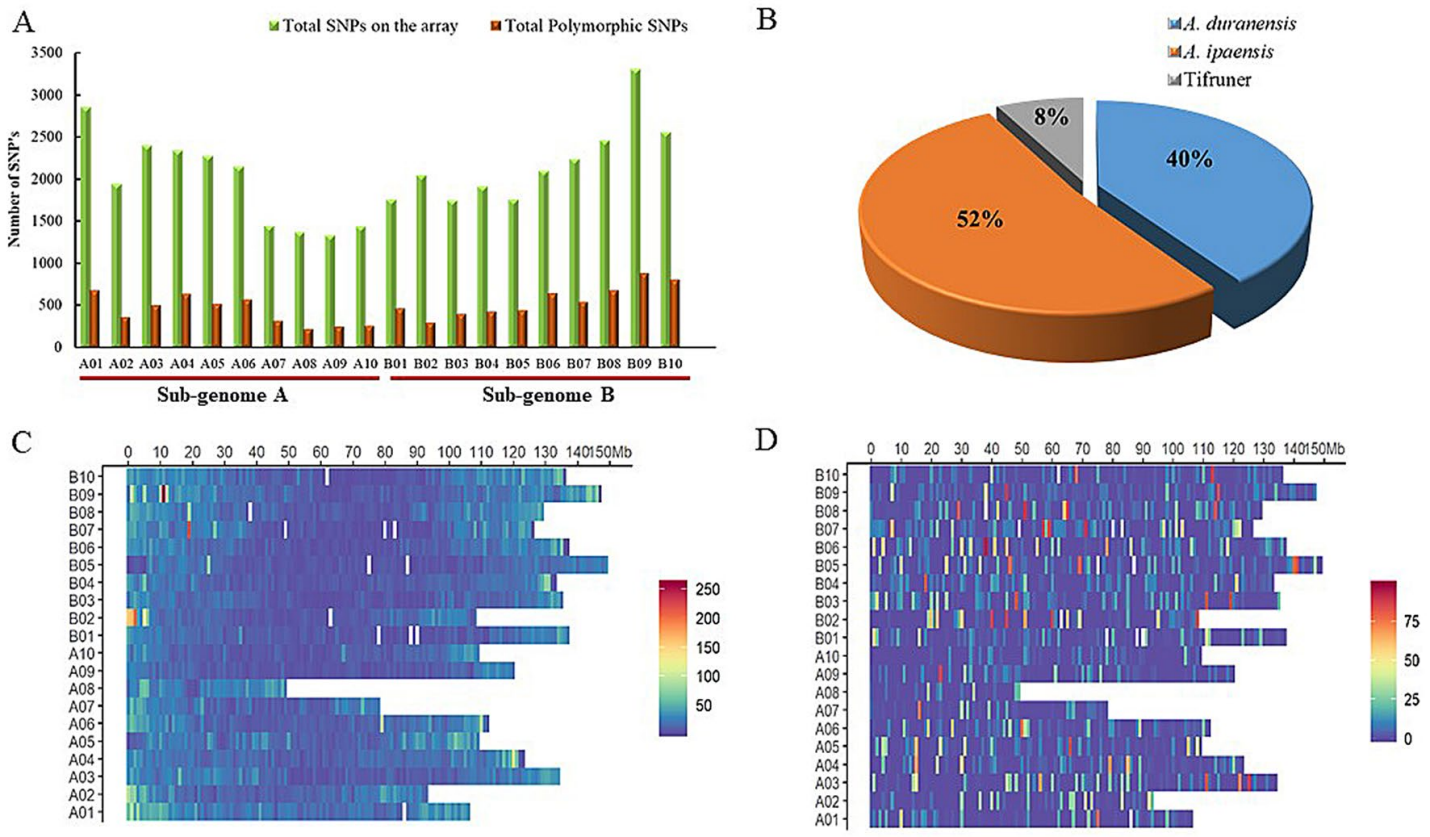
(**A**) Pseudomolecule-wise SNP distribution on the array and the distribution of polymorphic SNPs in the ‘Korean Set’ of genotypes, (**B**) Species-wise representation of SNPs (%), (**C**) Heat map of genome-wide distribution SNP calling pattern and genome density, (**D**) Heterozygosity distribution of genotype used for analysis (deep blue: A Homo; mid Blue: Hetero; light Blue: B Homo; white: blank).

A total 96 Korean set peanut genotypes from different countries (Table S1) were genotyped using the high-density SNP array ‘Axiom_*Arachis*’ 48 K SNP array. A total of 47,837 SNPs were initially obtained for these 96 Korean peanut genotypes based on SNP calling (Fig. 1A). Genome-wide distribution of SNP calling pattern, genome density, and the genotype heterozygosity used in analysis are shown in Fig. 1C,D. The distribution of these SNPs was accessed according to the pseudomolecule-reference genomes (A and B). Out of the 47,837 SNPs, 19,554 SNPs and 21,876 SNPs were derived from A subgenome and B subgenome, respectively. The remaining SNPs were from *A. hypogaea* cv. *Tifrunner* genome (Fig. 1A, Tables S3, and S4). The SNPs derived from each subgenome (A and B) were obtained based on 48 K Thermo Fisher ‘Axiom_arachis2’ SNP microarray annotation information (DNAlink Inc Seoul, South Korea) (Table S3). Species-wise representation of SNPs on the ‘Axiom_*Arachis’* array and their polymorphisms in the ‘Korean set’ genotypes were: *A. duranensis* (40.0%), *A. ipaensis* (52.0%), and *Tifruner* (8.0%) (Fig. 1B). After excluding SNPs with a missing rate > 10%, a minor allele frequency of < 0.05, and heterozygous call rate > 10%, final 'Poly High Resolution' (PHR) polymorphisms including 9947 SNPs were used for genetic analysis (Fig. 1A). Similarly, US mini-core genotypes along with Korean set genotypes designated as Merge set were used in genetic analysis using 4475 SNP loci obtained after filtering data. For US mini core, chip data were obtained from the PeanutBase (https://peanutbase.org/home). Further SNP genotype data of US mini core were filtered based on the shared or common Affymetrix ID (SNP site) between (US-mini core) and Korean set (96 genotypes) used for the analysis as the merge set (Table S1 and S2).

### Genetic diversity analysis of Korean set and Merge set

Evaluation of genotypic data of 96 Korean set genotypes showed a major allele frequency (MAF) of 0.837, a polymorphic information content (PIC) of 0.375. The expected heterozygosity (He) values ranged from 0.020 to 0.500 (mean value: 0.229, while the observed heterozygosity (Ho) values ranged from 0.00 to 0.979 (mean value: 0.048). The genetic diversity of the Merge set showed a MAF of 0.998, and a PIC of 0.375, the He values ranged from 0.005 to 0.499 (mean value: 0.333, while the Ho values ranged from 0.00 to 0.845 (mean value: 0.061) (Table 1). Results of genetic diversity indicated that neither the Korean set nor the Merge set of genotypes was very diverse. F-statistics (F_is_) of both sets (Korean and Merged) showed a high inbreeding coefficient (Table 1). Such results are expected as the peanut is known to be a highly self-pollinated crop. Genotypes used in this study might have been developed based on repetitive selection. Consequently, a high inbreeding coefficient of the relationship was expected.

**Table 1:** Summary of genetic diversity statistics of Korean set data (96 genotypes) and Merge set data (207 genotypes) of peanuts (MAF, major allele frequency; He, expected heterozygosity; Ho, observed heterozygosity; PIC, polymorphism information content; F_is_, F-statistics, inbreeding coefficient of an individual relative to subpopulations).

	MAF	H_e_	H_o_	PIC	F_is_
**Korean genotypes**					
Max	0.990	0.500	0.979	0.375	1.000
Median	0.885	0.201	0.000	0.182	1.000
Min	0.500	0.020	0.000	0.020	− 0.958
Mean	0.837	0.229	0.048	0.191	0.883
**Merge genotypes**					
Max	0.998	0.499	0.845	0.375	1.000
Median	0.679	0.434	0.010	0.341	0.977
Min	0.500	0.005	0.000	0.005	− 0.697
Mean	0.722	0.333	0.061	0.260	0.817

Besides, the relationships between the Korean set and the Merge set of genotypes were further investigated using IBS allele-sharing values. The MDS plot revealed that genome-wide IBS pairwise distances for Korean set and Merge set genotypes were 0.42–1.02 and 0.48–1.03, respectively (Figures S1A and S1B). Ten pairs of genotypes from the designated sets with maximum and minimum IBS values are presented in Table S7. The IBS-based genetic distance matrix in the Korean set and the Merge set of genotypes was calculated using the TASSEL version 5.2.39. (https://tassel.bitbucket.io/). The genetic distance ranged from 0.42 to 0.99, with an average value of (0.90) for the Korean set (Table S7). The lowest genetic distance (0.42) was observe between genotype GanghwaColl and Toalson in the Korean set. Likewise, the genetic distance ranged from 0.48 to 0.93 with an average of 0.99 for the Merge set. The lowest genetic distance (0.48) was observe between genotype TwungChungMuZhuVun and PI337399 in the Merge set (Table S7).

### Population structures of the Korean set and the Merge set

**Figure 2:**
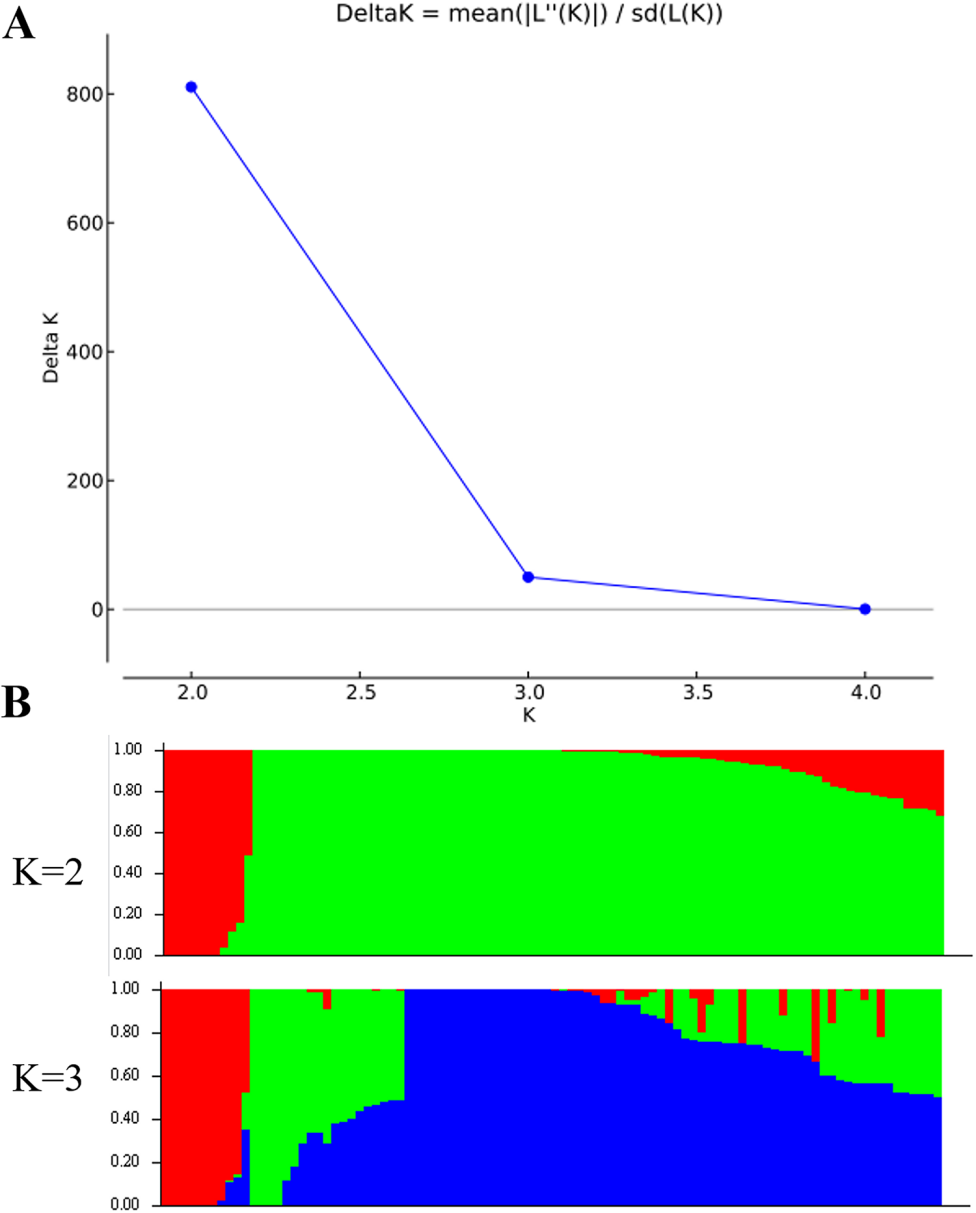
Population structure analyses of 96 peanut genotypes in the Korean set based on high-resolution SNPs assessed by STRUCTURE (http://pritch.bsd.uchicago.edu/structure.html). (**A**) Determining delta *K* values for different numbers of populations (*K* = 2) or 3 clusters (*K* = 3). (**B**) Bar plot for the K value = 2 or 3. The population structure analysis was performed with admixture 1.3^43^. Color segments represent different groups inferred by STRUCTURE analysis.

**Figure 3:**
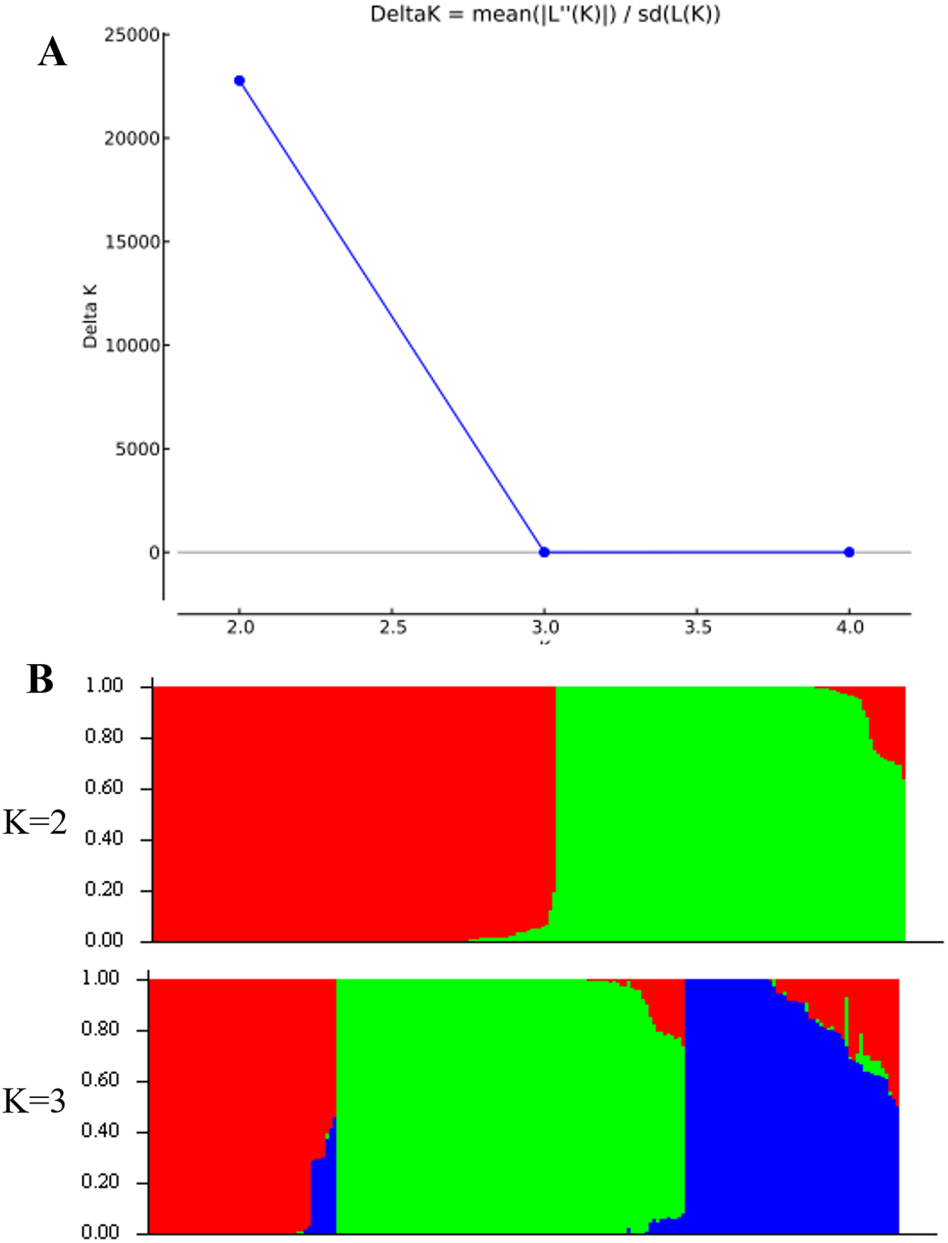
Population structure analyses of 207 genotypes in the Merge set based on the high-resolution 4475 SNPs assessed by STRUCTURE (http://pritch.bsd.uchicago.edu/structure.html). (**A**) Determining delta *K* values for different populations (*K* = 2) or 3 clusters (*K* = 3). (**B**) Bar plot for the K value 2 or 3. Population structure analysis was performed using admixture 1.3^43^. Color segments represent different groups inferred by STRUCTURE analysis.

To further determine subpopulations and the genetic relatedness among genotypes, we performed a STRUCTURE analysis (Figs. 2, 3). The population structure of the Korean and the Merged set of genotypes of peanuts was inferred based on 9947 SNPs and 4475 SNPs respectively using STRUCTURE 2.3.4. Software. Evaluation of genotypic data of Korean set genotypes showed the highest ΔK value (810.773) was observed when the value of K was 2 (Fig. 2A). These results suggested that the Korean set of peanut genotypes should be categorized into two groups (GI and GII) (Fig. 2B). Analysis with a K value of 3 was also performed to further explore the population structure (Fig. 2A). When K 3, at the second-largest value (ΔK = 50.114), the genotypes were divided into three groups (GI, GII, and GIII) (Fig. 2B). At K = 2, 81 genotypes fall in the GI group, whereas, 15 genotypes fall in the group GII (Table S1). Similarly, the population structure of the Merge set of genotypes showed maximum K value (22,778.77) at K2 (Fig. 3A), followed by K3. Based on the representative population structure, the merged set of genotypes could be divided into two groups (GI, GII) at K = 2, and further at K = 3, this set of genotypes were segregated into three groups (GI, GII, and GIII) (Fig. 3B). At K = 2, 152 genotypes and 55 genotypes fall in the GI, and GII groups respectively (Table S2). Overall, we found a mixed pattern of separation of all the genotypes and a lack of distinct separation with subspecies, botanical variety, lineage, or origin.

### Phylogenetic analysis

**Figure 4:**
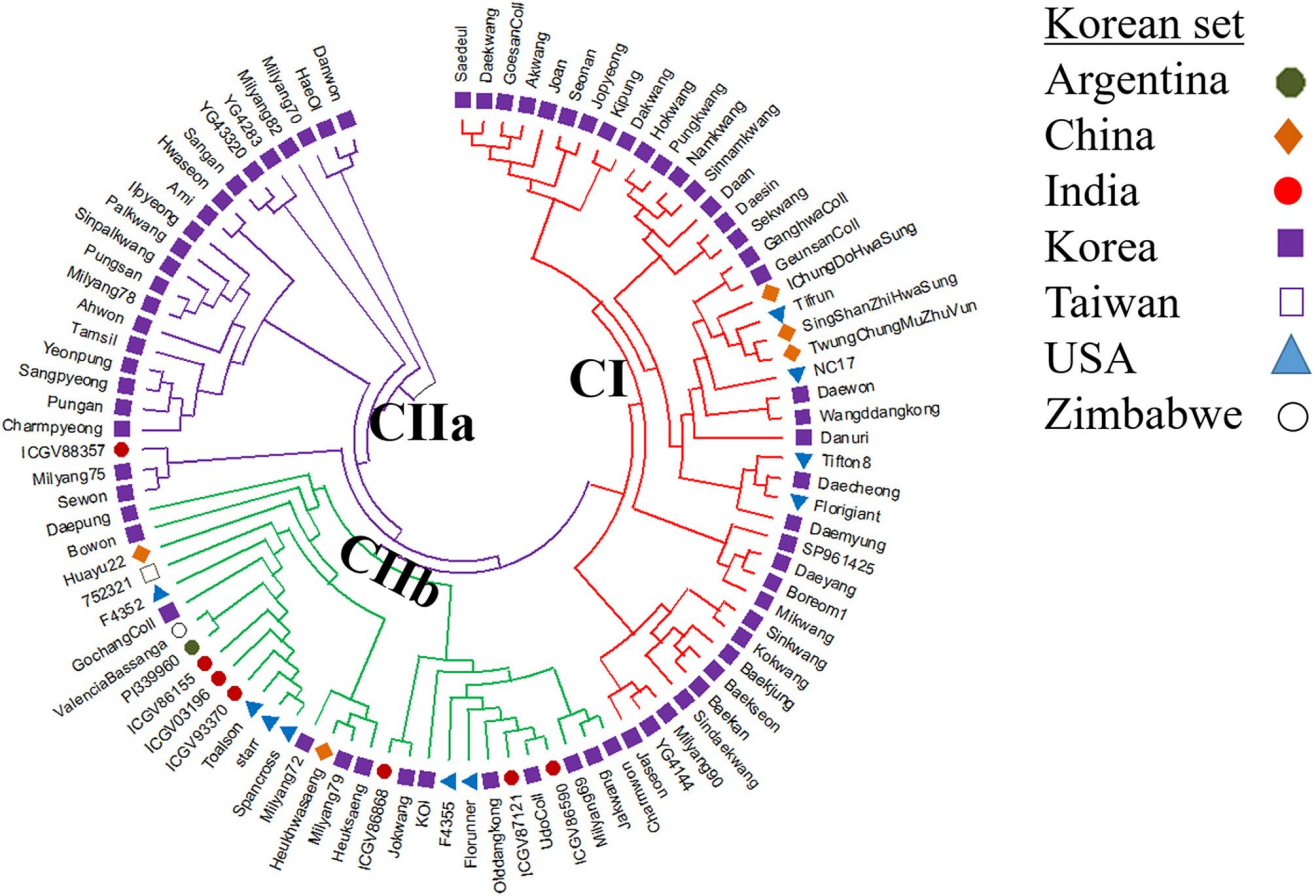
Phylogenetic tree of the Korean set of peanut genotypes (n = 96) constructed using MEGA X software (http://www.megasoftware.net/.) with a neighbor-joining method^44^. These genotypes were clustered into two major clusters. The second cluster was subdivided into CIIa and CIIb, with each colored branch representing respective clusters (CI, red; CIIa, purple; CIIb, green). The leaf node symbol represents the respective country mentioned in the legend.

**Figure 5:**
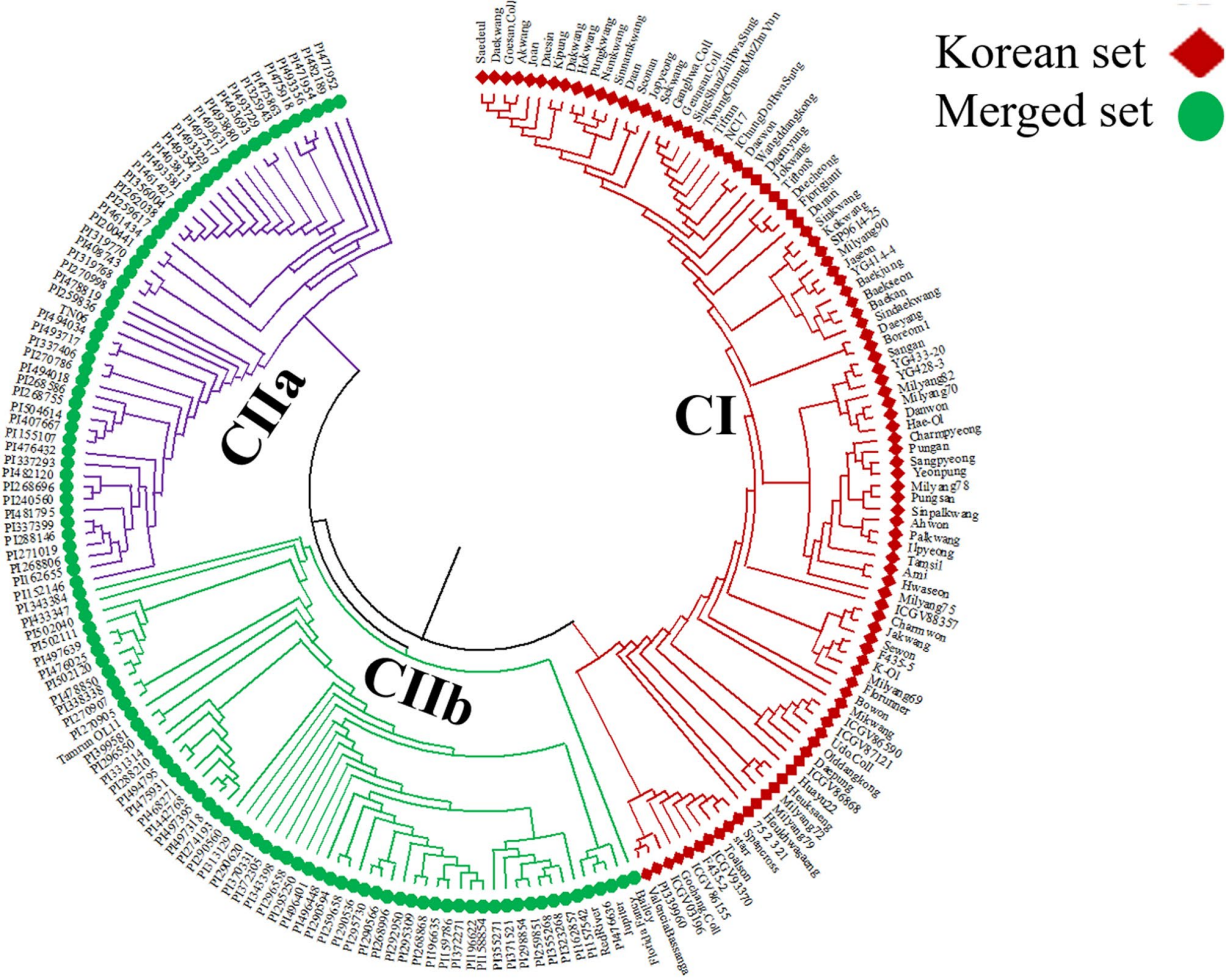
Phylogenetic tree of Merge set of peanut genotypes (n = 207) constructed using MEGA X software (http://www.megasoftware.net/.) with the neighbor-joining method^44^. These genotypes were clustered into two major clusters. The second cluster was subdivided into CIIa and CII, with each colored branch representing respective cluster (CI, red; CIIa, purple; CIIb, green).

A phylogenetic tree comprising 96 peanut genotypes in the Korean set was constructed based on 9947 high-resolution SNPs obtained after filtering (Fig. 4). These 96 peanut genotypes were divided into two clusters (CI and CII). The second cluster was subdivided into CIIa and CIIb. The CI cluster consisted of 43 genotypes, whereas the second cluster was manually divided into subclade I (n = 23) and CIIb (n = 30) genotypes (Fig. 4) based on the hierarchically clustered genotype profiles. Cluster CIIa comprised 22 Korean origin genotypes and only one genotype from India, whereas cluster CIIb comprised 13 of 30 genotypes with Korean origin. Based on the phylogenetic tree, Korean peanut genotypes might have been introduced from not only India and China, but also from other parts of the world like USA, Argentina, Taiwan, and Zimbabwe. Korean genotypes mainly belonging to cluster CIIb might have been directly introduced from different parts of the world (Fig. 4). A phylogenetic tree was also constructed for the Merge set of 207 genotypes having 4475 SNPs (Fig. 5). The Merge set of genotypes were also classified into two major clusters in the NJ phylogenetic tree. The CI cluster comprised 96 genotypes, whereas the second cluster was manually divided into subclades based on the hierarchically clustered genotype profiles as CIa and CIIb comprising 111 genotypes (Fig. 5). Although the mini core US genotypes belonged to different countries (Table S2), they formed a distinguish cluster (CIIa and CIIb) from the Korean set genotypes cluster (CI). Cluster CIIa comprised 50 genotypes belonging to three different botanical varieties (*fastagiata*, *hypogaea*, *vulgaris*) from diverse parts of the world (Table S2). Whereas, cluster CIIb comprised 61 genotypes belonging to three different botanical varieties (*fastagiata*, *hypogaea*, *vulgaris*) dominated with 8 genotypes from Israel and 6 genotypes from India. The remaining genotypes were from different parts of the world (Table S2). However, genotype distribution was not observed based on categories of subspecies, botanical variety, or origin. Overall, results for both the data sets were found to be consistent with the model-based population structure at K = 2.

### Principal component analysis

**Figure 6:**
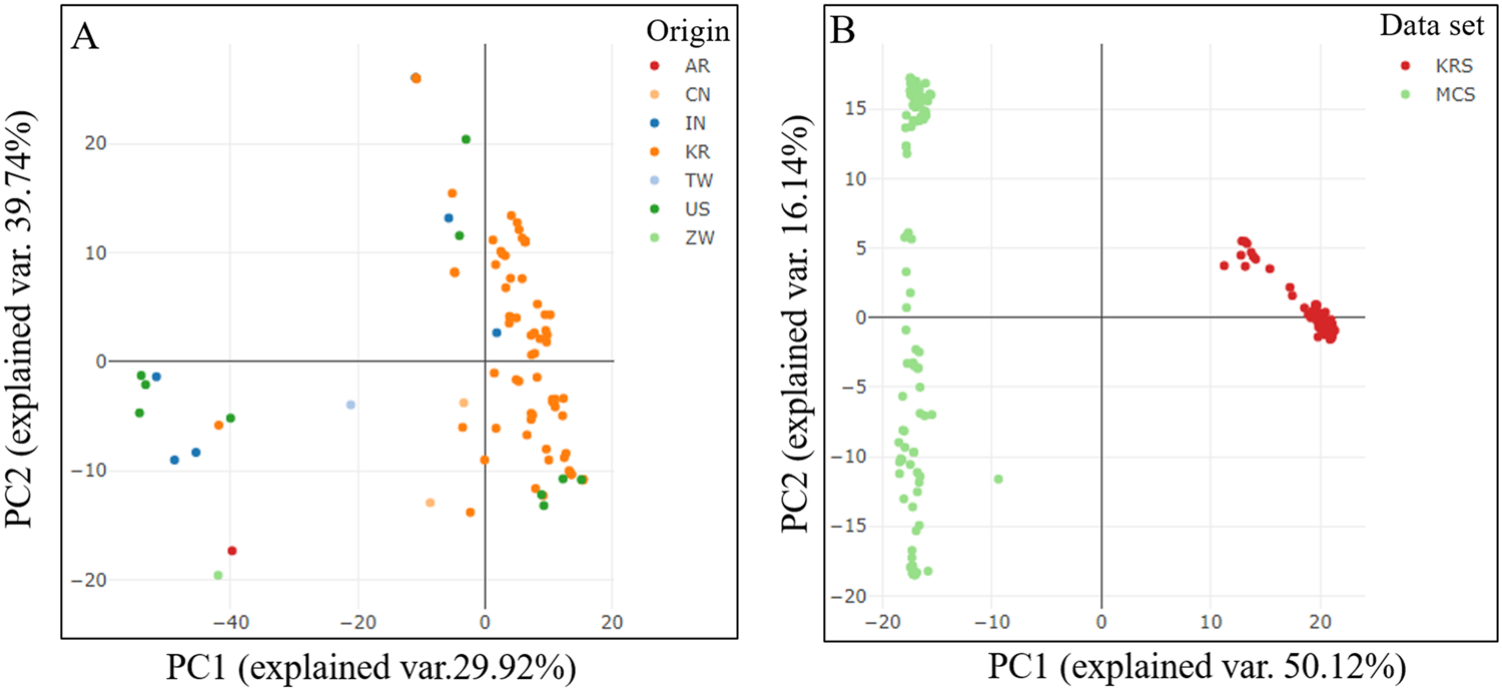
Principal component analysis (PCA) of Korean set and Merge set of peanut genotypes. (**A**) Korean set was classified according to the origin of genotypes (AR- Argentina, CN- China, IN- India, KR- Korea, TW-Taiwan, US- United State of America, and ZW-Zimbabwe). (**B**) Merge set classified according to the data set (KRS- Korean set of genotypes and MCS- Merged set of genotypes).

To further verify the clustering observed in the phylogenetic tree, PCA was conducted for the first two principal components (PC1 and PC2) of the Korean set of genotypes based on high-resolution SNP data. Scatter plots of the principal components displayed a variation of 29.92% for PC1 and 39.74% for PC2 (Fig. 6A). These scatter plots suggested that the Korean set of peanut genotypes were forming two main groups majority of Korean genotypes falling in the one group, consistent with clusters of the phylogenetic tree except for a slight overlap between some genotypes (Fig. 6A). Likewise, PCA was performed for the first two principal components of the Merge set of genotypes using 4475 SNPs. PC1 results explained 50.12% of the variance and PC2 explained 16.14% of the variance (Fig. 6B). The PCA plots in Fig. 6B correspond with Korean set genotypes in one cluster and Merge set genotypes into another cluster. These results suggest that the Merge set of peanut genotypes groups with different degrees of diversion among 207 genotypes and Korean set 96 genotypes more closely in other groups (Fig. 6B).

### Analysis of molecular variance (AMOVA)

The AMOVA results showed a population variation of 10.34% whereas individual samples within the population for the Korean set showed a difference of 89.66% (Table 2). Likewise, a population variation of 65.61% was observed whereas individual population samples of the Merge set showed a variation of 34.39% (Table 2). In the case of the Korean set, the genetic difference among subpopulations was low compared to the difference within individual subpopulations. Conversely, the genetic difference among subpopulations was high compared to the difference within individual subpopulations in the Merge set model-based population. These results suggested two subgroups, which were consistent with the population structure and phylogenetic tree analysis of both datasets.

**Table 2:** Analysis of molecular variance (AMOVA) among and within the populations of Korean set and Merge get of peanut genotypes (df: degree of freedom; %, Percentage).

Source of variation	*d.f.*	Sum of squares	Variance components	% of variation	*P *value
**AMOVA of model-based population (Korean set)**					
Among Population	1	3060.252	60.419	10.34	< 0.0000
Within population	94	49,246.425	523.898	89.66	
Total	95	52,306.677	584.317		
Fixation Index (FST)	0.103				
**AMOVA of model-based population (Merge set)**					
Among Population	1	149,248.507	1458.655	65.61	< 0.0001
Within population	203	155,192.190	764.494	34.39	
Total	204	304,440.698	2223.149		
Fixation Index (FST)	0.656				

### Development and validation of KASP markers

**Figure 7:**
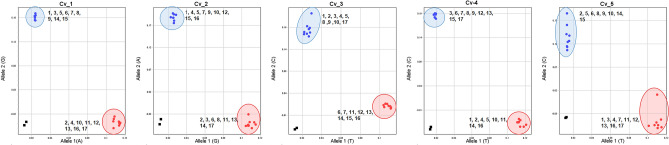
Validation of KASP marker and identification of 17 Korean peanut genotypes based on newly designed KASP markers. Results were visualized based on fluorescence signals emitted by the marker set (Cv_1—5). The number represents the designated genotype name (1: Daekwang, 2: Daepung, 3: Palkwang, 4: Akwang, 5: Baekjung, 6: Pungan, 7: Sangan, 8: Sinpalkwang, 9: Ami, 10: K-Ol, 11: Daan, 12: Heuksaeng, 13: Tamsil, 14: Sewon, 15: Haeol, 16: Milyang, 79, 17: Milyang, 75).

Based on Axiom SNP array data, we identified a few SNPs that could distinguish Korean origin peanut genotypes (n = 17) which are currently in use in the Korean peanut breeding program. These selected SNPs were used to develop the KASP primers. Further, all the designated genotypes (n = 17) were genotyped using these KASP markers to validate the results. Results of genotyping analysis yielded expected results as shown in Fig. 7 and Table S6. For example, the KASP marker CV_1 could distinguish the designated genotypes into two groups: Group 1 (Blue color), 1-Daekwang, 3-Palkwang, 5-Baekjung, 6-Pungan, 7-Sangan, 14-Sewon, 15-Hae-Ol, and 16-Milyang75; and Group 2 (Pink color), 8-Sinpalkwang, 9-Ami, 10-K-Ol, 11-Daan, 12-Heuksaeng, 13-Tamsil, 14-Sewon, 15-Hae-Ol, 16-Milyang79, and 17-Milyang75. Similarly, another KASP marker set (CV_2, CV_3, CV_4, and CV_5) distinguished these designated genotypes clearly from each other. These results indicated that these selected KASP marker sets could be utilized for the identification of varieties or for hybrid purity analysis. Further, these marker sets can be used in marker-assisted selection.

## Discussion

Advances in the genomic research of peanut are limited and advanced molecular techniques are underutilized to manage available germplasm and landraces. However, the elucidation of genetic relationships and genetic diversity among the germplasm or other breeding lines can lead to the precise use of genetic resources for crop improvement and for designing the breeding program^45, 46^. Recent developments in peanut genomic research, especially after the availability of its reference genome^7^ and different SNP arrays^37, 39^ for high throughput genotyping of peanuts, provide a great opportunity for accessing different genetic resources at the genetic level and for advancing the breeding program via multiple strategies.

SNP-based genotyping is widely used due to its accuracy, abundance, and high throughput. In this study, initially, a pseudomolecule-reference genome was identified based on the genomes of two diploid subspecies of cultivated peanut, *A*. *duranensis* (A genome) and *A*. *ipaensis* (B genome) as described previously^19, 37^. Furthermore, pseudomolecule-reference genome-wise distribution of SNP arrays and genome-wise polymorphic SNPs were identified (Fig. 1A, Table S3). This analysis was more or less similar to the previously reported studies^19, 37^. Further, the genetic diversity and the population structures of the Korean set of peanut genotypes (n = 96) were analyzed using 9947 high-resolution SNPs. At the same time, data related to mini-core US 111 genotypes were extracted from the public domain and combined with the Korean set of peanut genotypes to generate a Merge set. This Merged set was also used in the analysis of genetic diversity and population structure based on the 4448 polymorphic SNPs.. Results of genetic diversity analysis for the Korean set of genotypes showed *F*_*ST*_ values that suggested the genotypes in the Korean data set were more closely related. Moreover, Korean sets of genotypes seem to have a mixed lineage from parents that may belong to different geographical origins or gene pools.

Whereas, *F*_*ST*_ values of the Merge data set suggested that the US mini-core genotypes more diverse compared to Korean genotypes. Moreover, we observed a significant difference between the Korean data set and the mini-core data set (Fig. 5). As no Korean genotypes fall in CIIa or CIIb cluster (Fig. 5) and because the percentage of variation in the Merged set was 66% among populations (between Korean and mini-core genotypes) compared to 10% between-group CI and CII in the Korean set (Table 2). These results suggested that the Korean data set and the mini-core data of peanut genotypes were significantly diverse. Unlike the *F*_*ST*_ values, F-statistics (F_is_) of Korean set of genotype was slightly higher than merged set (Table 1). It was expected to have a higher inbreeding coefficient in Korean data set, however as we merged the Korean and mini-core genotypes and used only common SNPs between Korean data set and mini-core that could have led to higher F_is_ values in merged set as well. Commonly, the inbreeding coefficient (F_is_), determines the probability that two alleles at a given locus in an individual are equal by lineage from the shared ancestors of the two paternities. These results demonstrate that the mini core consisting of merged data have slightly less inbreeding compared to the Korean alone data set. Thus, it could be considered that the genetic diversity for Korean set peanut genotypes was low (Table 1) compared to the US mini-core genotypes. These results for genetic diversity were highly comparable to those of some recent studies conducted using SNPs markers for peanuts^19, 37, 47, 48^.

Structure within the population is best described when it is separated into two or three subgroups (K = 2 and K = 3). Particularly, the Korean set of peanut genotypes (n = 96) were categorized into two or three (GI, GII and GIII) groups (Fig. 2B). Likewise, 207 genotypes of the merged set were separated into two or three groups (GI, GII, and GIII) (Fig. 3B) at K = 2 and K = 3 respectively. However, STRUCTURE analysis for the Korean data set did not indicate correspondence with subspecies, botanical variety, or origin lineage and was observed as a mixed pattern of separation. Similar types of results were reported by the researcher in peanut mini core genetic analysis^20, 49^. Thus, this result indicated seeds of *Arachis hypogaea* L. might have been dispersed with wide geography from the center of origin.

Based on phylogenetic analysis, the Korean set of peanut genotypes (n = 96) were divided into two major clusters (CI and CII). CII was manually subdivided into CIIa and CIIb clades (Fig. 4). These Korean origin genotypes were distributed over all clusters CI, CIIa, and CIIb, with US runners/Virginias and US Spanish or Spanish parent-derived genotypes in CI, and CIIb clusters respectively (Fig. 4). More than 50.0% of Korean genotypes in the cluster CI had a close genetic distance with genotypes from the USA and China. Thus, these Korean peanut genotypes might have been derived from China and the USA. Other Korean genotypes were located in the CIIa cluster, showing 18.30% similarities with genotypes from different parts of the world (India, China, USA, Argentina, Taiwan, and Zimbabwe). Similarly, 31.0% of Korean genotypes from cluster CIIb showed similarities with a single Indian genotype, indicating that these genotypes belonging to this cluster might have been originated from India. Similarly, phylogenetic analysis for the Merge set of 207 genotypes showed two major groups (Fig. 5). As expected, Cluster CI (n = 96) genotypes belonged to the Korean set whereas clusters CIIa and CIIb comprising 111 genotypes were derived from the US mini-core set that formed a separate cluster (Fig. 5). In Fig. 5 all the Korean genotypes are in cluster CI, along with a few US Spanish origin genotypes whereas, cluster CIIa and CIIb are with the majority of genotypes belong to the mini core collection, and a few US runners. These results indicate that germplasm exchange between Korea and other parts of the world might have occurred. Different peanut genotypes in both sets (Korean and Merged) across the globe were separated into two major groups, although individuals in groups were mixed without any correlation with subspecies, botanical variety, or origin. However, the phylogenic results were in agreement with the results of the model-based population structure analysis at K = 2 for both the Korean set and the Merge set of peanut genotypes. Besides, the clustering and grouping patterns of genotypes of different origins could be explained precisely using an SNP array. They were comparable to those reported in previous studies^18, 19, 50–52^. Though the mini core genotypes were selected to exploit the genetic diversity in the peanut core and germplasm collection, however, we found a high extent of genetic resemblance among the genotypes which is predictable since peanut has typically known for low genetic diversity. Previously, in the GWAS study researcher reported a high level of similarities among the mini core accession^49^.

Moreover, the clusters derived from both sets of genotypes were further validated by PCA (Fig. 6). Results were consistent with relationships indicated by phylogenetic tree analysis and structure analysis. AMOVA results explained the differential variation between the Korean set and the Merge set of populations (Table 2). Population variation among the Korean set was 10.34%, whereas a difference of 89.66% was found in individual samples within the population of the Korean set (Table 2). Likewise, a variation of 65.61% was found among the populations of the Merged set and a variation of 34.39% was observed within the individual population samples of the Merge set (Table 2).

Overall, this study suggests that the high inbreeding coefficient and less variability in tested Korean set of genotypes. This might be instigated due to the self-pollination and repetitive selection of these peanut lines over the years during cultivar development and breeding process, which might have consequently reduced the genetic diversity. Therefore, higher similarity and low genetic variability were observed in tested Korean set of genotypes of the current study. This observation is in line with the pollination system and the history of peanut cultivation.

The increasing number of individuals and the routine use of population studies in genetics and breeding programs require flexibility and precision in genotyping methods. KASP marker assay is one such genotyping method that has emerged recently. It offers several advantages such as flexibility, robustness, multiplexing, cost-effectiveness, and rapid genotyping of small to large populations with hundreds of markers^53–55^. Thus, we developed KASP marker assays to validate and distinguish important pre-breeding genotypes using a unique SNP set that showed high-resolution calls on the Axiom 48 K SNP assay and polymorphism between selected genotypes. Although the number of markers used for validation was small, the viability and usefulness of these markers were analyzed by selecting fewer SNPs to distinguish Korean origin peanut genotypes (n = 17). Our results were similar to those using Axiom array genotyping, thus confirming and validating results of SNP array (Table S5). Validation using KASP markers suggested that a newly developed marker set could be utilized to identify varieties or analyze hybrid purity. In addition, these sets of markers could be used in marker-assisted selection or marker-trait association studies. Our results were consistent with previous studies reporting the application of KASP assays in legume species including peanuts^51, 53, 54, 56^.

## Conclusion

Results of this study showed a low genetic variation in Korean genotypes that might be caused by domestication, intentional selection, reduced population and genetic drift relation with the same gene pool, and breeding for some desirable traits. In the present study, genotype distribution was not found with the category of subspecies, botanical variety, or origin. However, this study revealed that the US mini-core set of genotypes were more diverse and with a negative or lower inbreeding coefficient than the Korean set of genotypes. This suggests that the Korean breeding scheme needs to widen the genetic base of breeding material and create new alleles or gene pool combinations by diversifying the current Korean breeding resources and increasing the breeding population. Besides, some genetically diverse genotypes might be useful for creating new trait combinations, developing a mapping population with desirable traits, improving crops, and generating new cultivars via appropriate breeding approaches. The present study also provides a number of high-resolution polymorphic SNP markers distributed in A and B subgenomes of peanut subspecies. They can facilitate the development of a new marker set for differentiation of varieties. Furthermore, these SNPs can be utilized to develop SNP-based genetic map, mapping applications, background selection, and substantial molecular breeding applications in the Korean peanut breeding program.

## Supplementary Information

Supplementary Figure.

Supplementary Information.

Supplementary Tables.
