# Supplementary figures and images for: Genetic diversity analysis of Korean peanut germplasm using 48 K SNPs ‘Axiom_Arachis’ Array and its application for cultivar differentiation
Source: Sci Rep. 2021 Aug 17;11:16630. doi: 10.1038/s41598-021-96074-4 (PMC8371136; doi:10.1038/s41598-021-96074-4)

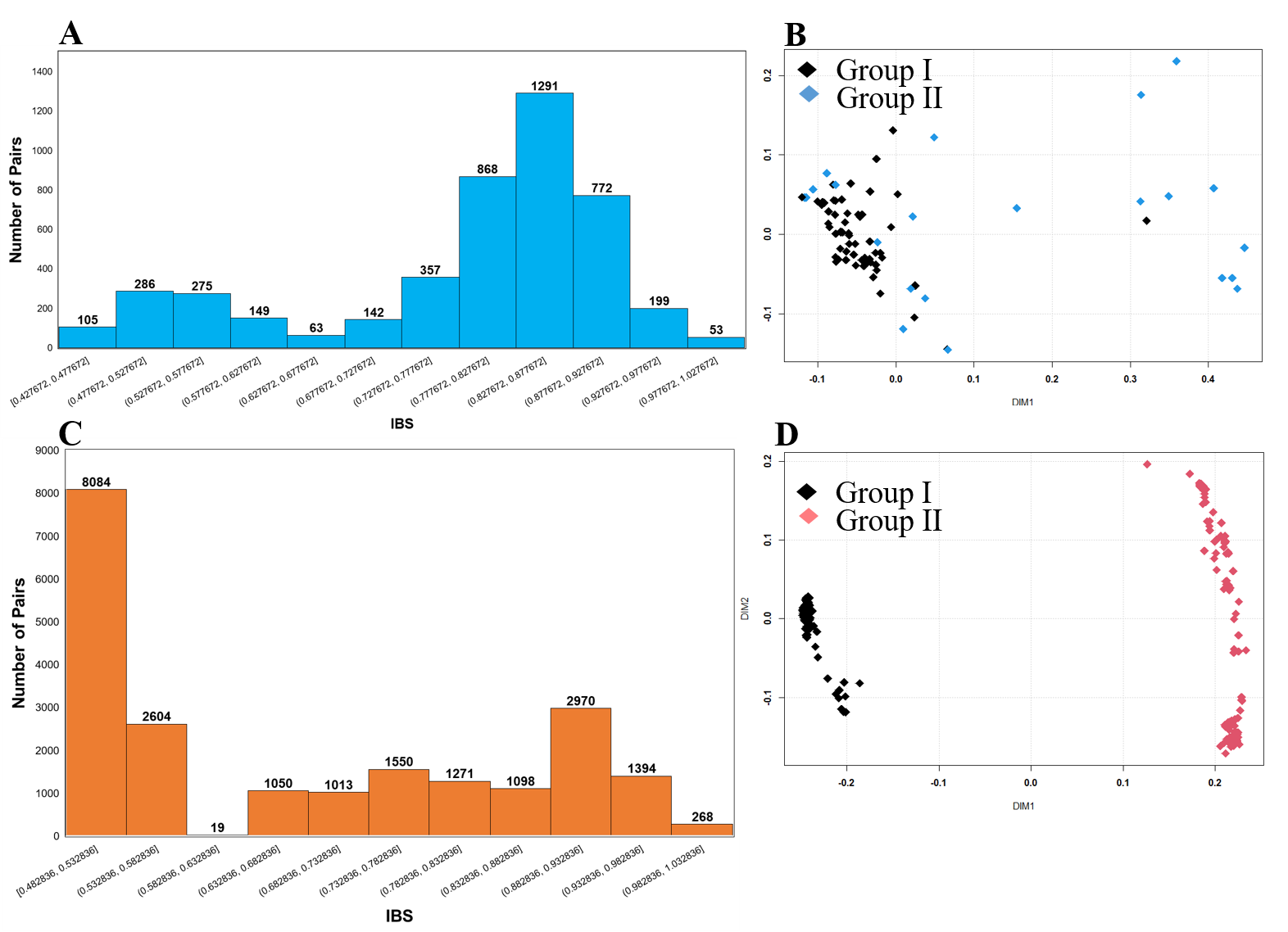

Supplement: Supplementary file 1 — Supplementary Figure. [file 41598_2021_96074_MOESM1_ESM.png]
