# Supplementary material for: Genetic diversity analysis of Korean peanut germplasm using 48 K SNPs ‘Axiom_Arachis’ Array and its application for cultivar differentiation
Source: Sci Rep. 2021 Aug 17;11:16630. doi: 10.1038/s41598-021-96074-4 (PMC8371136; doi:10.1038/s41598-021-96074-4)
Supplement: Supplementary file 2 — Supplementary Information. [file 41598_2021_96074_MOESM2_ESM.docx]

**legend for the supplementary figure**

**Figure S1.** Genetic diversity analysis based on identity by-state analysis (IBS) and visualisation by Multi-dimensional scaling (MDS). (A) Pair-wise IBS allele-sharing using the 9478 SNPs between 96 Korean Set genotypes were calculated using PLINK96 V1.90. (B) MDS plot representing the two groups in population. (C) Pair-wise IBS allele-sharing using the 4475 SNPs between 207 Merge Set genotypes were calculated using PLINK96 V1.90. (D) MDS plot representing the two groups in population.
